# The complete mitochondrial genome of *Lucilia shenyangensis* (Diptera: Calliphoridae)

**DOI:** 10.1080/23802359.2021.1947911

**Published:** 2021-07-15

**Authors:** Tingjun Chen, Xiangrong Li, Yong Wang

**Affiliations:** a Department of Forensic Science, School of Basic Medical Sciences, Central South University, Changsha, China; b Research Center for Special Medicine, Central South University, Changsha, China

**Keywords:** Mitochondrial genome, *Lucilia shenyangensis*, phylogenetic analysis

## Abstract

*Lucilia shenyangensis* Fan, 1965 (Diptera: Calliphoridae) is of potential importance in epidemiology, veterinary medicine, and forensic entomology due to their necrophilous habit and behaviors associated with mammals. In this study, we report the complete mitochondrial genome (mitogenome) of *L. shenyangensis*. The mitogenome is 14,989 bp in length, comprising 13 protein-coding genes (PCGs), two ribosomal RNAs (rRNAs), 22 transfer RNAs (tRNAs), and a non-coding control region. The arrangement of genes is identical to that of the ancestral metazoan. Nucleotide composition revealed a high A/T bias, accounting for 76.50% total mitogenome nucleotides (A 39.2%, G 9.6%, C 14.0%, T 37.3%). Phylogenetic analysis indicated that *L. shenyangensis* was clear separated from other blow flies and emerged as the sister lineage to the rest species from genus *Lucilia* (*L. illustris*, *L. sericata*, *L. coeruleiviridis,* and *L. porphyrina*). The mitogenome data of *L. shenyangensis* could facilitate further evolutionary genetic researches on blow flies.

Blow flies (Diptera: Calliphoridae) are distributed all over the world, which include over 1000 species from 150 genera described globally (McAlpine 1981). *Lucilia shenyangensis* Fan, 1965, belongs to the genus *Lucilia* of subfamily *Luciliinae* in Calliphoridae. It is widely distributed from the northwest (Ningxia province) to the northeastern region (Heilongjiang province) of China as well as Korean Peninsula, east coastal border region of Russia (Xu and Zhao 1996). *L. shenyangensis* that are attracted by carrion and garbage is of potential importance in epidemiology, veterinary medicine, and forensic entomology (Xu and Zhao 1996). In this study, we presented the complete mitochondrial genome (mitogenome) of *L. shenyangensis*. Mitogenome has become generally used for in-depth evolutionary and population genetic studies, which have been demonstrated to be able to refine the resolution of phylogenetic relationships among arthropods (Cameron 2014). In forensic entomology, mitochondrial DNA are often used for species identification. Mitochondrial genome research of blow flies could expand the available genetic data on dipteran, which greatly improves the accuracy of molecular marker-based species identification. Moreover, the currently used genetic markers still cannot completely distinguish some close species within blow flies, and the further expansion of complete mitogenome database will help to find more suitable molecular markers.

In this study, we collected the adult specimens of *L. shenyangensis* attracted by pig liver in June 2019 from Beijing city (39°48′N, 116°28′E), China. After being identified by traditional morphological characteristics (Figure 1) (Xu and Zhao 1996), the specimens were sacrificed by freezing and stored at −80 °C in Dr. Guo’s Lab (Changsha, Hunan, China) with a unique code (CSU20190628). Total DNA was extracted using QIAamp Micro DNA Kit (QIAGEN Biotech CO., Ltd, Hilden, Germany) from the thorax muscle of an adult fly according to the manufacture’s instruction. The mitogenome sequencing of *L. shenyangensis* was performed on an Illumina HiSeq 2500 Platform (Illumina, Inc., San Diego, CA, USA), and then the initial annotation of the mitogenome was carried out with MITObim v1.9 and (https://github.com/chrishah/MITObim) and SOAPdenovo v2.04 (Hahn et al. 2013). The gene boundaries were verified by MITOS2 Web Server (http://mitos2.bioinf.uni-leipzig.de/index.py) (Bernt et al. 2013). The gene annotation results were compared with available Calliphoridae mitogenomes to ensure the accuracy using MEGA X for gene alignment (Kumar et al. 2018), and the transfer RNAs (tRNAs) were verified with other dipteran insects by tRNAscan-SE Search Server v1.21 (Lowe and Chan 2016).

**Figure 1.**
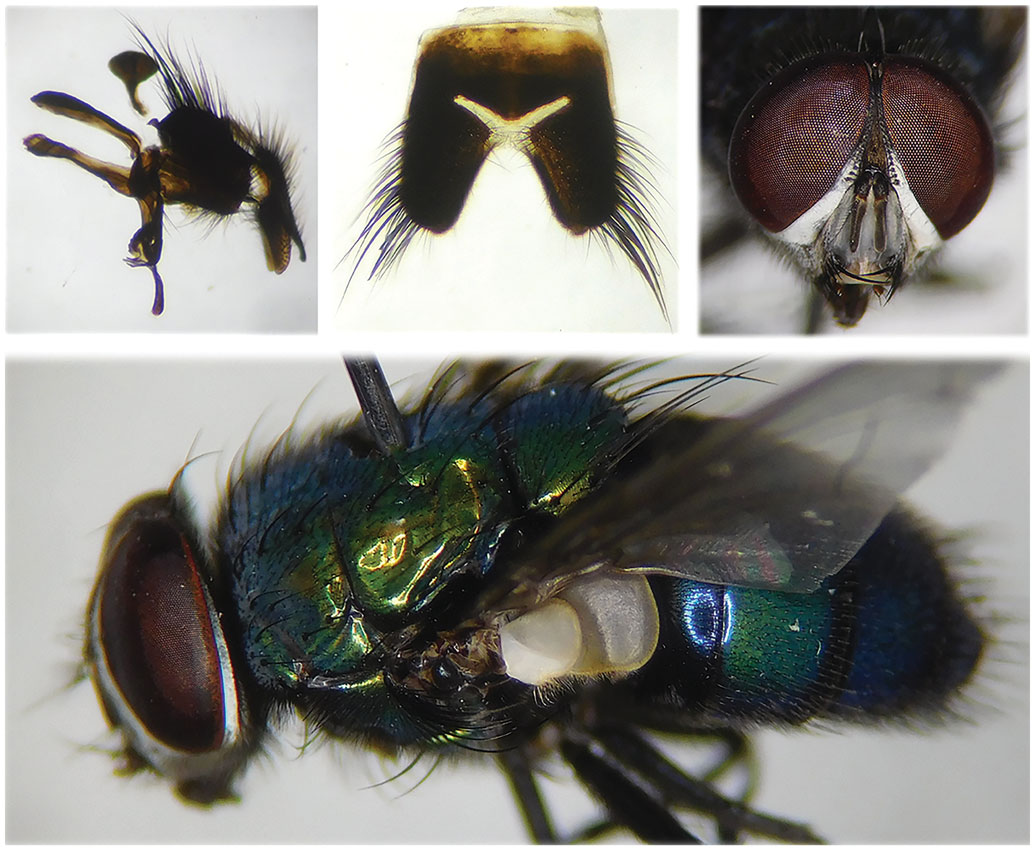
Morphological characteristics of adult *Lucilia shenyangensis*.

The complete mitogenome of *L. shenyangensis* is 14,989 bp in length (GenBank accession No. MW446947), containing 13 protein-coding genes (PCGs), two ribosomal RNAs (rRNAs), 22 transfer RNAs (tRNAs), and a non-coding control region. The distribution order of all elements is identical to that of the ancestral metazoan (Cameron 2014). Nucleotide composition revealed a high A/T bias, accounting for 76.5% (A 39.2%, G 9.6%, C 14.0%, T 37.3%) of total nucleotides. Phylogenetic trees of *L. shenyangensis* and other 12 calliphorids species were constructed using the maximum likelihood (ML) method with the GTR substitution model based on the 13 PCGs, and *Sarcophaga africa* (Diptera: Sarcophagidae) was used as an outgroup (Figure 2). All these sequences were aligned using MAFFT version 7 software (Katoh and Standley 2013). The ML analysis was performed using IQ-TREE v1.6.12 with ultrafast likelihood bootstrap set to 1000 replicates (Nguyen et al. 2015). The phylogenetic relationships indicated that *L. shenyangensis* was clearly separated from other blow flies and emerged as the sister lineage to the rest species from genus *Lucilia* (*L. illustris, L. sericata*, *L. coeruleiviridis,* and *L. porphyrina*). Therefore, the mitogenome data reported in this study provides additional genetic information for further evolutionary relationships studies on blow flies.

**Figure 2.**
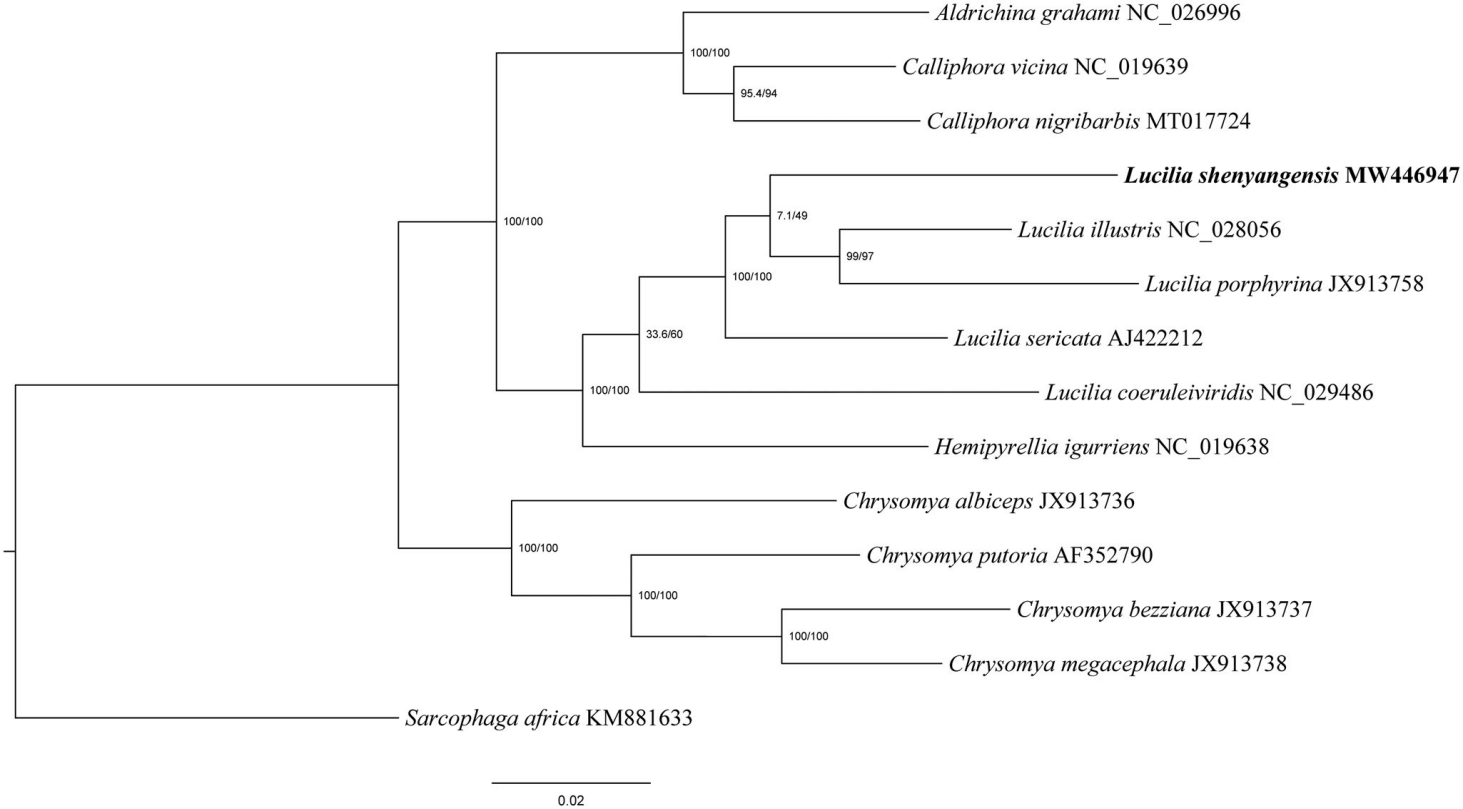
Phylogenetic trees of *Lucilia shenyangensis* with 12 calliphorids species based on 13 protein-coding genes by the maximum likelihood (ML) method. *Sarcophaga africa* (Diptera: Sarcophagidae) was selected as an outgroup.

## Data Availability

The assembled mitochondrial genome is available on NCBI at https://www.ncbi.nlm.nih.gov/nuccore/MW446947 (accession No. MW446947). Associated BioProject, SRA, and BioSample accession numbers are PRJNA693991, SRR13512606, and SAMN17491226, respectively. (https://www.ncbi.nlm.nih.gov/bioproject/PRJNA693991; https://www.ncbi.nlm.nih.gov/sra/SRR13512606; https://www.ncbi.nlm.nih.gov/biosample/SAMN17491226)
